# Biological function simulation in neuromorphic devices: from synapse and neuron to behavior

**DOI:** 10.1080/14686996.2023.2183712

**Published:** 2023-03-10

**Authors:** Hui Chen, Huilin Li, Ting Ma, Shuangshuang Han, Qiuping Zhao

**Affiliations:** a Heart Center of Henan Provincial People’s Hospital, Central China Fuwai Hospital, Central China Fuwai Hospital of Zhengzhou University, Zhengzhou, P. R. China; b Henan Key Laboratory of Photovoltaic Materials, Henan University, Kaifeng, P. R. China

**Keywords:** Neuromorphic computing, artificial intelligence, memristor, synapse, neuron

## Abstract

As the boom of data storage and processing, brain-inspired computing provides an effective approach to solve the current problem. Various emerging materials and devices have been reported to promote the development of neuromorphic computing. Thereinto, the neuromorphic device represented by memristor has attracted extensive research due to its outstanding property to emulate the brain’s functions from synaptic plasticity, sensory-memory neurons to some intelligent behaviors of living creatures. Herein, we mainly review the progress of these brain functions mimicked by neuromorphic devices, concentrating on synapse (i.e. various synaptic plasticity trigger by electricity and/or light), neurons (including the various sensory nervous system) and intelligent behaviors (such as conditioned reflex represented by Pavlov’s dog experiment). Finally, some challenges and prospects related to neuromorphic devices are presented.

## GRAPHICAL ABSTRACT

**Figure:**
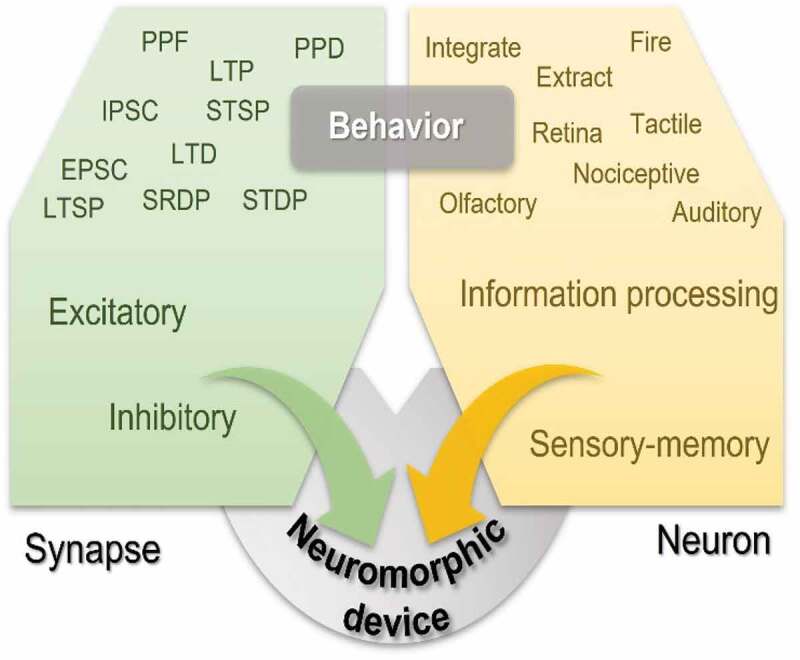

## 1. Introduction

With the continuous development of the Internet, the online world demands that the computer should have more storage and faster processing for the big-data. However, these abilities are quickly approaching their theoretical limit for the storage and processing. Because the modern computing systems are built on von Neumann architecture, memory wall (a physical separation) is formed between memory and processor, which hinds the speed for information processing. Moore’s law shows that the electronic devices should be getting smaller and smaller in order to increase their storage density, but the physical size limit and high energy consumption have not made it go down any further. Therefore, it is an urgent requirement to develop new devices beyond the Moore’s law and von Neumann bottleneck [1,2].

To address these issues, researchers have kept a watchful eye on the human brains, which have incomparable advantages over the modern computing systems: ultralow energy consumption (10–20 W) and processing-in-memory [3]. Hence, brain-inspired computing, also called neuromorphic computing, has emerged to duplicate these brain-functions. Nowadays, research about neuromorphic computing basically includes three aspects: neuromorphic device (artificial synapses and neurons), neuromorphic circuitry (device networks) and neuromorphic algorithm (learning rules and training methods) [4]. In the human brain, there are about 10^15^ synapses, and about 10,000 synapses consist a neuron, which enables brain to achieve information processing and memory function [5]. Therefore, the key thing for neuromorphic computing is to develop electronic devices with synaptic and neural functions. At first, silicon-based complementary metal-oxide-semiconductor (CMOS) transistors are used to mimic the synapse behaviors, but multiple transistors are always required to perform one synaptic function [4,6]. Meanwhile, they often suffer from volatility, moderate scalability and inefficient synaptic operation. Recently, memristors are directly used to simulate the synaptic and neural functions [7–9]. Compared with conventional CMOS transistors, one memristor can well realize the synaptic and neural functions, and integrate memory and logic operation in one unit device. The memristor (combination of the word ‘memory’ and ‘resistor’) is defined as a two-terminal system with resistive switching (RS) effect, which is first proposed by Chua in 1971 and in which the variable and non-volatile resistance are closely relied on the history of operated voltage and current [10–12]. But in many years afterwards, no one has succeeded in getting either a useful physical model or an example of a memristor. Until 2008, when HP developed a TiO_2_-based device with adjustable resistance and memory characteristics, the prelude of simulating synaptic plasticity by using memristor was opened [13]. Since then, various types of memristors and neuromorphic devices have appeared in abundance. Therein, they have developed from two-terminal to three- and multi-terminal devices; the used materials for these devices have been from traditional oxide materials to the novel materials, including quantum dots, halide perovskites and two-dimension (2D) materials; the biological functions have mimicked synaptic plasticity at first and then developed to simulate neuronal activity and some biological behaviors. However, most of the review articles are on materials and devices in the field of memristors and neuromorphic devices. Therefore, it is necessary to summary biological function simulation in these devices, which is more conducive to the research and development of new memristors and neuromorphic devices.

Herein, we devote to reviewing the recent progress for biological function simulation in memristors and neuromorphic devices. First, we introduce the biological basis in memristors and neuromorphic devices. Then, the biological functions for synapse, neuron and biological behaviors are presented for the recent progress in these smart devices. Finally, future research challenges in memristors and neuromorphic devices are discussed.

## 2. Principles of nervous system

The nervous system in vertebrates (e.g. human beings) has up to 86 billion neurons, and 99.9% of them are distributed in the brain, which supports numerous intelligent functions including memorizing and forgetting, learning, and decision-making [14–16]. Billions of neurons interweave via synapses to form a complex network in the brain, in which information is transmitted between two neurons through synapses. Intelligent is built up in the brain by this way. To develop the brain-like devices (i.e. memristors and neuromorphic devices), it is very necessary to understand the structures and working mechanism of biological neurons and synapses.

### 2.1. Biological neurons

**Figure 1.**
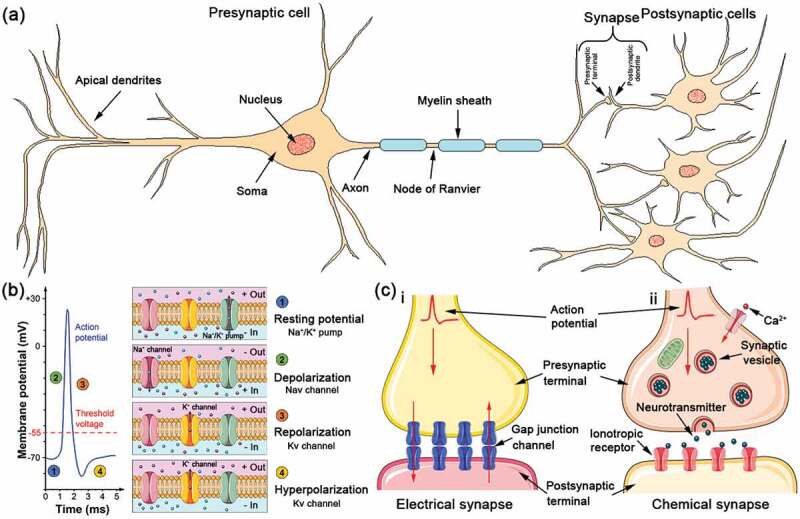
Schematic diagram of the fundamental structure of the biological neuron model (a), the molecular basic of generating an action potential including resting potential, depolarization, repolarization and hyperpolarization (b) and the two main types of synapses: chemical synapse and electrical synapse (c).

Neuron is the signaling unit of nervous system. A typical neuron has four morphologically defined parts: (1) soma (or cell body), (2) dendrites, (3) axon, and (4) presynaptic terminal (Figure 1(a)). Therein, soma can synthesize the neurotransmitters and integrate the electrical signals from the dendrites to control the transmission of action potential, which is the metabolic center of a neuron that contains the nucleus (provides the genes) and endoplasmic reticulum (synthesizes the proteins). The soma usually gives rise to two types of cytoplasmic protrusions: several short dendrites and one long tubular axon. Dendrites that branch out in tree-like fashion are the main apparatus to receive the incoming signals from other nerve cells and play a critical role in filtering and integrating these signals to determine whether to fire a signal or not, but they cannot amplify these input signals. Dendrites can assist self-neurons to monitor instructions from neighboring neurons [17]. Compared with dendrites, axons have a larger length, and some can extend over 2 m within the body. In addition, most axons in the central nervous system are very thinner (0.2–20 μm in diameter) compared with the diameter of the soma (≥50 μm). For typical axons, many of them are insulated by some sheathes of fatty myelin that are regularly interrupted at gaps called the nodes of Ranvier. The axons are considered as the transmitting element of a neuron that can deliver electrical signals to other neurons through presynaptic terminal, where the synapses are formed with the postsynaptic dendrites. Usually, a neuron has one soma, one axon, many dendrites and presynaptic terminals, in which the basic function of one neuron is to exchange information by receiving, integrating, conducting and exporting information (electrical signals).

The electrical signals, called action potentials, are initiated at the initial segment of axon and propagate down the axon to the nerve terminals at speeds of 1 to 100 m/s. Action potentials have four properties: initiation threshold, all-or-none nature, conduction without decrement and refractory period. Due to these properties, action potentials display the change with four segments: resting potential, depolarization, repolarization, and hyperpolarization (Figure 1(b)) [18]. Resting potential is that the membrane potential is in a relatively steady state. In this state, the concentrations of Na+ and K+ on both sides of the cell membrane remain basically unchanged. Net current flowing through the membrane is zero, so the neuron is silent in this case. Once the membrane potential is potentiated, voltage-gated sodium (Nav) channel is activated to enter the membrane and a rapid influx of Na+ is formed under the potential gradient. At this time, the membrane potential can change from negative to positive, which suggests entering the depolarization process. When the membrane potential reaches its peak, K+ starts to flow out of the cytomembrane in order to neutralize the excessive potential, leading to repolarization of the membrane potential. After that, K+ continues to outflow in order to restore the normal membrane potential, which is called hyperpolarization [2,16]. Therefore, the physiological process of depolarization is dominated by Nav channel, while the repolarization and hyperpolarization processes are regulated by voltage-gated potassium (Kv) channel. Due to these, the action potentials are transported in/among neurons.

### 2.2. Biological synapses

Synapse is the specialized site where one neuron communicates with another, which consists of a presynaptic membrane, synaptic cleft, and postsynaptic membrane. The average neuron forms several thousand synaptic connections and receive a similar number with its neighboring neurons. Synaptic transmission is basic to the brain functions, such as perception, learning and memory. Two modes of synaptic transmission are found in all neurons: electrical and chemical. Based on this, the synapses are divided into electrical and chemical synapses (Figure 1(c)).

Electrical synapse is the gap junction, a special way of cell-to-cell linkage, which makes the action potential direct transmission between cells (Figure 1(c-i)). For this synapse, the synaptic cleft is very small, only several nanometers. In the presynaptic and postsynaptic membrane, there are some connexons that are made up of connexins. Two connexons form a gap junction channel, a non-gate control channel, which allows some small molecules of water-soluble substances and ions to pass through. When the action potential is generated in a neuron, the local current based on ionic current can be directly stimulated and transmitted to another neuron through the gap junction channel. By this way, the action potential is propagated from neuron to neuron. From this, the electrical synapse has lots of outstanding features, such as low resistive, rapid and bidirectional propagation. Different from electrical synapse, chemical synapse depends on the neurotransmitters to accomplish the information transfer from neuron to neuron (Figure 1(c-ii)). In chemical synapse, there are more mitochondria and a large number of vesicles, in which the latter is also called synaptic vesicle with 20–80 nm diameter and high contains concentrations of neurotransmitters such as acetylcholine or amino acid transmitters, catecholamine transmitters and neuropeptide transmitters. When the action potential is transmitted to the presynaptic terminal of a neuron, the presynaptic membrane depolarizes. After the depolarization exceeds the threshold value, Ca^2+^ channel is activated and Ca^2+^ enters into the axoplasm of the terminal from the outside of the cell. The increase of Ca^2+^ can trigger the efflux of synaptic vesicles and cause the quantized release of neurotransmitters. Meanwhile, excess Ca^2+^ in the axoplasm is transported outside through Na+-Ca^2+^ reverse transporter in order to its normal concentration in the presynaptic terminal. Once the neurotransmitters are released, they can enter into the synaptic cleft and reach the postsynaptic membrane by diffusion. Ultimately, these neurotransmitters can act on the ionotropic receptor and control the permeability of certain ions. When certain ions enter the postsynaptic terminal, if the terminal occurs depolarization, the signal is called excitatory postsynaptic potential (EPSP). In this process, the excitatory neurotransmitters act on the ionotropic receptors in the postsynaptic terminal to open the specific ion channels (Na+ and K+). The net inward current is generated because Na+ influx of is greater than K+ outflow, in turn, lead to the depolarization of the postsynaptic terminal. On the contrary, inhibitory neurotransmitters are released from the presynaptic terminal to act on the ionotropic receptors, and then open the Cl- channel to generate the outward current that hyperpolarizes the postsynaptic membrane. In this case, it is called inhibitory postsynaptic potential (IPSP). By this way, the information is transmitted to the next neuron by releasing neurotransmitters. The distinguishing between electrical and chemical synapses is listed in Table 1. However, the chemical synapse is the majority of synapses in the human brain.

**Table 1.** Distinguishing properties of electrical and chemical synapses [21].

Type of synapse	synaptic cleft	cytoplasmic continuity between neurons	Ultrastructural components	Agent of transmission	Synaptic delay	Direction of transmission
Electrical	4 nm	Yes	Gap junction	Ion current	Virtually absent	Bidirectional
Chemical	20–40 nm	No	Presynaptic vesicles and active zones, postsynaptic receptors	Chemical transmitter	Significant at least 0.3 ms, usually 1–5 ms or longer	Unidirectional

### 2.3. Neural plasticity

In the biological system, neural plasticity, including synaptic plasticity and nonsynaptic plasticity, is the ability to change the synapse or neuron properties, which gives the functions of learning and memory to our brains [19,20]. Nonsynaptic plasticity is mainly the intrinsic plasticity, involving the persistent modification of a neuron’s intrinsic electrical properties by regulating the voltage-dependent ion-channels. Intrinsic plasticity is closely related to many different forms of learning, e.g. spatial learning, classical conditioning, odor and fear conditioning. However, the function of intrinsic plasticity in brain is still unknown. By contrast, synaptic plasticity is the most prominent and extensively studied form of neural plasticity.

Synaptic plasticity refers to changes in the efficiency of synaptic transmission from a physiological standpoint, which makes postsynaptic response last for a certain time from tens of milliseconds to several weeks or longer [21,22]. According to the duration time, synaptic plasticity can be classified as two types: short-term and long-term synaptic plasticity (STSP and LTSP). As short-term synaptic dynamics, STSP often lasts for several minutes or shorter, which comes in three forms: facilitation (paired pulse facilitation, PPF), augmentation (post-tetanic potentiation, PTP) and depression (short-term depression, STD). Therein, facilitation often lasts for hundreds of milliseconds or shorter, while augmentation and depression can achieve to several minutes. For PPF, when two action potentials act on the synapse in a short interval (often tens of milliseconds), the prior action potential acts on presynaptic terminal to increase Ca^2+^ in presynaptic terminal, but the later has arrived at synapse before Ca^2+^ returns to the normal state. It leads to more neurotransmitters to be released and generate larger EPSP. PTP occurs after a short string of high-frequency action potentials that can make Ca^2+^ in the axoplasm to temporarily accumulate [23]. Similar to PPF and PTP, STD will occur in some synapses after repeated action potentials. Another synaptic plasticity is LTSP that can last several hours even for weeks [24]. Long-term potentiation (LTP) and long-term depression (LTD) are the two forms, corresponding to the persistent enhancement and decrement in synaptic strength. Studies show that LTP occurs after executing a high-frequency electrical stimulation for several seconds, while LTD is activated by a low-frequency (1 Hz) stimulation for longer time (10–15 min) [25]. Spiking-timing-dependent plasticity (STDP), another typical form of LTSP, can bring about LTP and LTD according to the relative timing between pre- and postsynaptic activation, which is important to achieve time-dependent computing tasks for the brain [26]. Different from STSP, LTSP is ascribed to increasing Ca^2+^ in postsynaptic terminal instead of presynaptic terminal. However, the physiological mechanisms of LTSP vary at different synapses.

Furthermore, many other forms of synaptic plasticity, such as spiking-rate-dependent plasticity (SRDP), associative and nonassociative learning, synaptic scaling and synaptic redistribution, are also indispensable for information processing and neuromorphic computation [27–29]. It is worth emphasizing here that there is much unknown about the cognition of our brains, and the related brain science and neuroscience studies are very essential to reveal the nature of synaptic plasticity and understand the working mechanisms of biological nervous systems.

## 3. Artificial synapses

Biological synapses are the most key parts in neural networks of our brains to achieve the transmission and memory of information. Artificial synapses, trying to mimic the function of biological synapses, have also been widely studied as a new type of electronic device. From this perspective, artificial synapses depend on the conductance (or resistance) change in electronic circuits instead of regulating weight. Depending on the principle of conductance, various types of materials (e.g. resistive, phase change, magnetic and ferroelectric materials) and devices (e.g. two-terminal memristors and multiterminal neuro-transistors) are extensively used in artificial synapses, which have been emphasized in many previous papers [6,30–35]. In this section, we focus on the biological features to different types of artificial synapses.

### 3.1. Electrical synapses

Synaptic plasticity is the significant feature of biological synapse, and is also the key to empowering the brain to learn and memory. To develop the artificial synapses, the top priority is to realize synaptic plasticity in the electronic device. Memristor is one of the earliest studied artificial synapses and is also the most extensive and mature one now. In memristor, the resistance changes with the electrical current flow passing through. Once the current has stopped, it can keep the previous value until the reverse current is applied to restore it to its original state. Due to this, the memristor is usually used to simulate the synaptic plasticity under the specific electrical pulses.

#### 3.1.1. Excitatory and inhibitory

**Figure 2.**
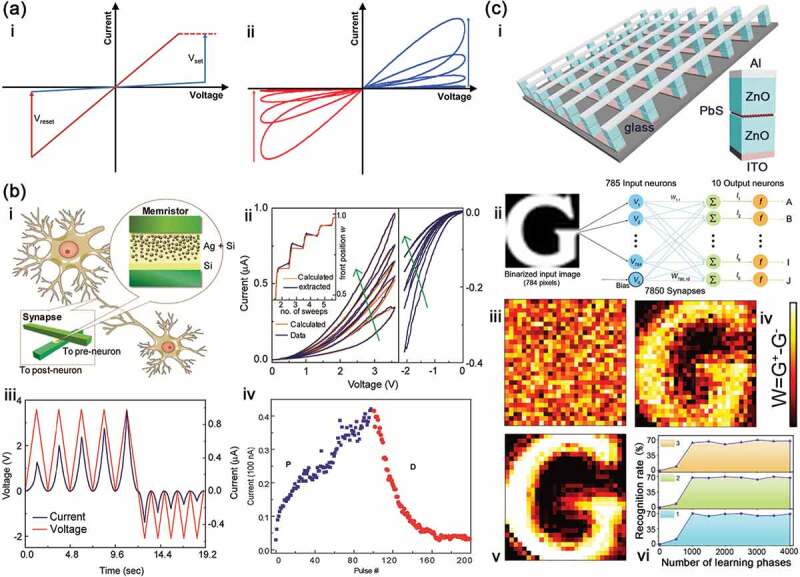
(a) Schematic diagram of the typical I–V curves for RRAM (i) and memristor (ii). (b) the Ag-gradient memristor characteristics and its application as a synapse: i, schematic diagram of this memristor as synapse between two neurons; ii, I-V characteristics of the memristor; iii, the current and voltage changes versus time for the device; iv, excitatory and inhibitory characteristic of this device. Reproduced with permission from Reference [36]. Copyright 2010 American Chemical Society. (c) the memristor based on ZnO/PbS hybrid heterostructure and its application as a synapse: i, schematic diagram of the device array; ii, schematic illustration of simulated ANN; mapping images of synaptic weights connected to output letter ‘G’ for iii without training, iv after 500 times and v after 1000 times training; vi, recognition rate as a function of number of learning phases for iii to v. Reproduced with permission from Reference [37]. Copyright 2019 Elsevier.

Memristor is developed on the basis of the resistive random access memory (RRAM), and their characteristic curves are shown in Figure 2(a). For RRAM, resistive switching occurs under voltage/current sweeps and constant voltage/current pulses when the device is changed between low resistance state (LRS, ON state) and high resistance state (HRS, OFF state) (Figure 2(a-i)), whereas that of memristor is called pinched hysteresis loop like oblique ‘8’ glyph, and it is worth noting that the unilateral curve showed an increasing/decreasing change under the positive/negative voltage sweeps and pulses (Figure 2(a-ii)). These increasing and decreasing changes are similar to the potential and depression behaviors of biological synapses, so memristors can be used to simulate excitatory and inhibitory synapse. More than 10 years ago, researchers have simulated these behaviors of biology synapses by using memristors. In 2010, Lu et al. [36] reported a two-terminal nanoscale silicon-based memristor device by sputtering Ag and/or Si on the tungsten electrode to form regions with different concentrations of Ag nanoparticles in Si films (Figure 2(b-i)). This device has a similar structure with biological synapse, where top (chrome/platinum) and bottom electrodes like presynaptic and postsynaptic membranes, and Si (Ag) films as synaptic cleft. The Ag nanoparticles can gradually form Ag conductive filament and vice versa. Based on this switching mechanism, the output current displays monotonically increasing and decreasing changes under voltage sweep and pulse number (Figure 2(b-ii,iii)). By controlling the electrical pulses, the potentiating and depressing plasticity has been simulated successfully in this device. Since this report, many articles have been reported to simulate this plasticity [9,38–41]. For example, Zeng et al. [9] prepared a homogenous bilayer memristor with a structure of W/HfO_y_/HfO_x_/Pt to emulate synaptic plasticity, wherein the oxygen vacancy (V_O_) in HfO_x(y)_ is similar to the Ca^2+^ profile in biological synapse to control the output signals. Due to this, the reading current of the device has the increasing and decreasing changes after ±5 V pulses for writing and −2.5 V pulses for reading. Han et al. [38] introduced a flexible organic synaptic transistor with C_60_ floating-gate and poly(methyl methacrylate) (PMMA) tunnelling dielectric layers. Due to charge trapping of C_60_, the channel conductance can increase or decrease quantitatively so that excitatory and inhibitory features of biological synaptic have been emulated successfully.

The excitatory and inhibitory features of these synapses can be used for pattern recognition in artificial neural network (ANN) [37,40–42]. We have reported a two-terminal memristor with hybrid heterostructure (Figure 2(c-i)) [37]. This device exhibits an increasing output current under the positive pulses, while a decreasing current under the negative pulses. Based on these properties, the ANN can perform supervised learning with the not-Modified National Institute of Standards and Technology (notMNIST) small images (Figure 2(c-ii–vi)). After certain phases training, the mapping images display the clear output letter ‘G’, and the recognition rates have reached to 68 ± 6%. Zhang et al. [42] reported a low-voltage memristor based on an ultrathin 2D PdSeO_x_/PdSe_2_ heterostructure to use in the neuromorphic computing. In the paper, ANN network, including 400 input neurons, 100 hidden neurons and 10 output neurons, has a high recognition accuracy of 93.4% after training based on the excitatory and inhibitory features of PdSeO_x_/PdSe_2_ memristor. Meanwhile, they also demonstrated that the PdSeO_x_/PdSe_2_ memristor can be better applied at convolutional image processing. Remarkably, the accuracy is closely associated with linearity and symmetry of conductance states for the devices. High linearity and symmetry are beneficial to improve the accuracy. For this reason, Keene et al. [43] exploited an ionic floating-gate memory array based on a polymer redox transistor connected to a conductive-bridge memory. The device displays near-linear conductance under the positive and negative pulses, and maintains a high signal-to-noise ratio as well. After training, the accuracy is as high as 98%.

#### 3.1.2. STSP and LTSP

**Figure 3.**
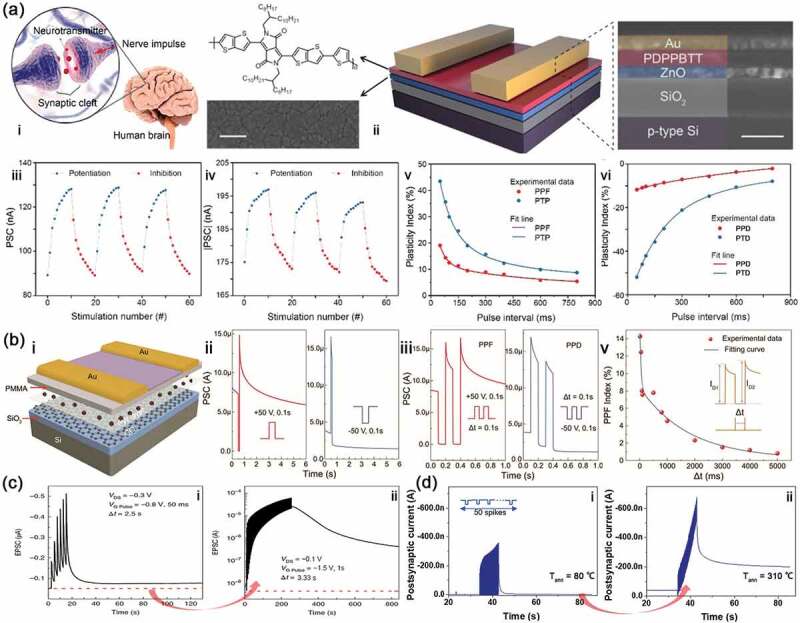
Electrical synapses and the plasticity. (a) the artificial synapse based on PDPPBTT/ZnO heterojunction and its application as a synapse: schematic diagram of the structure comparison between biological synapse (i) and this artificial synapse (ii); Circulation of potentiation and inhibition under the electron-enhancement (iii) and hole-enhancement mode (iv); PPF and PTP (v), PPD and PTD (vi) changes (calculate from equation 1 and 2, respectively) as a function of pulse interval of paired presynaptic spikes. Reproduced with permission from reference [47]. Copyright 2020 American Chemical Society. (b) the memristor based on reduced graphene oxide (RGO) and its application as a synapse: i, schematic diagram of the memristor structure with Si/SiO_2_/PW-RGO/PMMA/pentacene/Au source-drain; ii, EPSC triggered of single presynaptic spike; iii, PPF and PPD features of the memristor; iv, PPF changes (calculate from equation 1) as a function of pulse interval (δt) of paired presynaptic spikes. Reproduced with permission from reference [48]. Copyright 2018 Wiley-VCH. (c) STSP is transformed to LTSP by increased spike number. Reproduced with permission from reference [49]. Copyright 2019 Nature. (d) STSP is transformed to LTSP by increased temperature. Reproduced with permission from reference [50]. Copyright 2019 Elsevier.

Based on excitatory and inhibitory characteristics, two-form plasticity can be simulated easily in artificial synapses. Therein, STSP, playing a key role in the biological sensory neural system, neuromorphic computation or ANN, is deemed to modulate the dynamic synaptic efficacy during the transient transmission process (from milliseconds to seconds) through low-pass or high-pass temporal filtering functions [44,45]. In biological systems, PPF and PTP are two types of STSP in the excitatory synapse, which describe a fact that excitatory postsynaptic current (EPSC) is aroused by the second and the tenth pulse, respectively [46]. Correspondingly, paired-pulse depression (PPD) and post-tetanic depression (PTD) are for inhibitory synapse, and inhibitory postsynaptic current (IPSC) aroused by the second and the tenth pulse, respectively. In the artificial synapse, PPF, PTP, PPD and PTD can be obtained from the following equations [37]:

(1) $$PPF\;or\;PPD=\;\left(I_{2}-I_{1}\right)/I_{1}\;\times \;100\%$$

(2) $$PTP\;or\;PTD=\;\left(I_{10}-I_{1}\right)/I_{1}\;\times \;100\%$$

where I_1_, I_2_ and I_10_ are the currents recorded under the first, second and tenth pulse stimulus, respectively. In addition, these plasticity characteristics are closely related to the interval time (*t*) between each pulse, which can be fitted to the double exponential function:

(3) $$Y=C\;+\;A_{1}exp\left(-t/T_{1}\right)\;+\;A_{2}exp\left(-t/T_{2}\right)$$

where C is a constant, A_1_ and A_2_ are the initial facilitation magnitudes, and T_1_ and T_2_ are the relaxation times. For example, Wang et al. [47] reported an ambipolar transistor based with poly(2,2-(2,5-bis(2-octyldodecyl)-3,6-dioxo-2,3,5,6-tetrahydropyrrolo[3,4-c]pyrrole-1,4-diyl)dithieno[3,2-b]thiophene-5,5-diyl-alt-thiophen-2,5-diyl) (PDPPBTT)/ZnO heterojunction, displaying hole-enhancement and electron-enhancement modes in the transistor (Figure 3(a)). In the two modes, EPSC are both aroused, and the PPF, PTP, PPD and PTD are successfully simulated that they are compatible with the biological synapse. STSP often displays delay the short delay process, in which EPSC is greater than that of the initial state while IPSC is the opposite. As shown in Figure 3(b), a flash memory is used to mimic the STSP by Chen et al. [48] After applied positive and negative voltage pulses, PSC displays excitatory (higher current level than the base line) and inhibitory (lower current level than the base line) because electrons and holes are injected to the gate, respectively. Meanwhile, PPF and PPD property can be mimicked under applying, respectively, two +50 V and two −50 V consecutive presynaptic spikes in this memory device.

We often find a fact that we need to read a poem several times before we remember it, which is because the LTSP is formed in our brains after repeating the same stimulus many times. For the brain-inspired devices, STSP should be transformed to LTSP so that the information is stored in electronic devices for a long time. Therefore, researchers actively simulate this transition in the artificial synapse devices through changing the pulse state or some external conditions [49–54]. Lenz et al. [49] prepared an artificial synapse with vertical electrolyte-gated organic transistor (Figure 3(c)). After six pulses (−0.8 V, 50 ms) with an inter-spike interval of 2.5 s, the PSC returns quickly to a stable value that is slightly larger than the initial state, whereas EPSC, triggered by 73 pulses (−1.5 V, 1 s) with an inter-spike interval of 3.33 s, displays a long delay process and after several hundreds of seconds it has a much greater value than the initial one. Seo et al. [50] reported an organic synaptic transistor that emulates universal synaptic behaviors of both brain and peripheral nervous systems (Figure 3(d)). In this, the device also displays the LTSP characteristic after treating by a high annealing temperature (T_ann_) with the obvious long-delay phenomenon. In biological system, STSP can make human brains forget easily, which requires relearning to change it into LTSP in order to keep the memory for a long time or even forever. For the artificial synapse, it also needs the LTSP to form the non-volatile memory.

**Figure 4.**
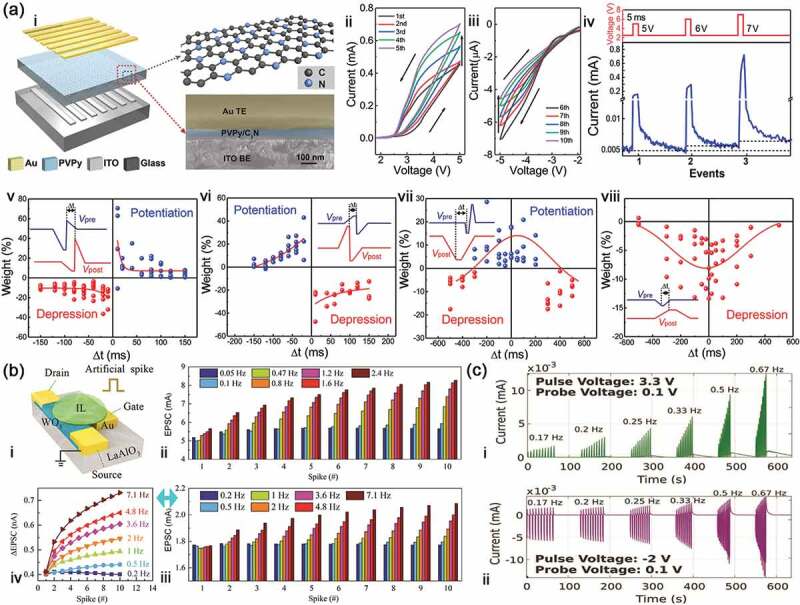
Electrical synapses and the plasticity. (a) the memristor based on C_3_N/PVPy composite film and its application as a synapse: i, schematic diagram of the memristor structure; I-V characteristics of the memristor under consecutive positive (ii) and negative (iii) voltage sweeps; iv, EPSC responses to the pre-synaptic pulses with various pulse voltages; STDP implementation in the C_3_N/PVPy-based artificial synapse with Asymmetric Hebbian (v), asymmetric anti-Hebbian (vi), symmetric Hebbian (vii) and symmetric anti-Hebbian (vii) learning rule. Reproduced with permission from Reference [57]. Copyright 2019 Elsevier. (b) WO_3_-based transistor and its application as a synapse: i, schematic diagram of three-terminal synaptic transistor; ii and iii SRDP characteristic of this synaptic transistor; iv current change corresponding to iii. Reproduced with permission from Reference [58]. Copyright 2018 Wiley-VCH. (c) SRDP characteristic under positive (i) and negative (ii) voltage pulse. Reproduced with permission from reference [59]. Copyright 2021 Wiley-VCH.

Once the STSP is triggered from the STSP, PPF and PPD also become LTP and LTD, respectively. These synaptic functions, displaying intrinsically frequency dependent, are also considered as the basis of learning and memory. Therein, STDP is regarded as one of the most basic protocols of these and significantly affects the long-term synaptic modification, in which the synaptic weight is regulated by the relative time (∆*t*) between the presynaptic and postsynaptic spikes [55]. For typical STDP, the synaptic weight (∆*w*) will increase and display the LTP characteristic if presynaptic spike pulls ahead postsynaptic one. In contrast, if presynaptic spike lags behind postsynaptic one, the synaptic weight (∆*w*) will decrease and display the LTD characteristic. With the development of neuroscience, four STDP forms have been in the biological system containing asymmetric Hebbian STDP, asymmetric anti-Hebbian STDP, symmetric Hebbian STDP and symmetric anti-Hebbian STDP [56]. For these, the weight updates can be expressed as the following equations:

(4) $$\Delta w_{a}\;=\;Aexp\left(-\Delta t/\tau \right)\;+\;\Delta w_{0}$$

(5) $$\Delta w_{s}\;=\;Aexp\left(-\Delta t^{2}/\tau^{2}\right)\;+\;\Delta w_{0}$$

where ∆*w*_*a*_ and ∆*w*_*s*_ represent asymmetric and symmetric STDP, respectively, ∆*w*_*0*_ is the constant representing a nonassociative component of the synaptic change, A is the scaling factor and *τ* is the time constant. Zhou et al. [57] reported an artificial synapse with the adjustable synaptic plasticity based on solution-processed 2D C_3_N/polyvinylpyrrolidone (PVPy) (Figure 4(a)). The device displays the obvious memristor characteristics and the tunability between STSP and LTSP. Most importantly, these four STDP forms are successfully simulated by regulating presynaptic and postsynaptic spikes. Similarly, Yu et al. [60] prepared a restickable oxide neuromorphic transistor based on the biodegradable material of chitosan. These STDP learning rules are also demonstrated, which include Hebbian STDP, anti-Hebbian STDP, symmetrical STDP and visual STDP.

SRDP is another important protocol of learning and memory. In neurobiology, biological memory is based on synaptic weight that is achieved by tuning the frequency of stimulation. Unlike STDP, SRDP relies on presynaptic spikes with different frequency to cause the variation of potentiation and depression. For example, Yang et al. [58] prepared a planar transistor using WO_3_ as semiconductor channel and ionic liquid as dielectric layer (Figure 4(b)). For this artificial synapse, gate pulse voltages are deemed to be presynaptic spikes, and a small voltage is acted on the drain and source to read the channel current. In this case, the device can trigger EPSC in the WO_3_ channel. On this basis, they used two kinds of positive voltage pulses with different frequency to achieve obvious SRDP, in which the EPSC peak value and their relative change can enhance with increasing the pulse frequency. Ebenhoch and Schmidt-Mende [59] reported a two-terminal memristor with TiO_2_ nanowire array as active layer, fluorine doped tin oxide (FTO) as bottom electrode and Au tip as top electrode. Positive or negative voltage pulses are acted on the electrode and each pulse is recorded with a small-based voltage of 0.1 V. The current value of peaks displays good frequency dependence, i.e. well SRDP (Figure 4(c)). During the process to simulate these plasticity behaviors, an important factor of energy consumption (E) must be taken seriously. The energy consumption can be calculated by the following equation:

(6) $$E=I_{peak}\times t_{d}\times V_{r}$$

where I_peak_, t_d_ and V_r_ represent the peak value of the PSC, the duration time of the pulse voltage, and the record or read voltage, respectively. Therefore, it is of great importance to develop the artificial synapse with fast pulse, low voltage and current [61].

### 3.2. Light synapses

Except electricity, light is also used to simulate the synaptic plasticity in the artificial devices. By utilizing light, two superiorities have been in these synapses: giving visual perception and reducing energy consumption [62–65]. In the biological system, more than 80% of information about our surroundings for human brain is acquired through visual perception [66]. In many cases, light can be used directly in the electrical synapses due to the photoelectric properties of materials to selectively simulate synaptic plasticity. In other words, different wavelengths of light show different differences in synaptic simulation, so light synapse can carry out the visual perception for the outside world. In another case, light modulation has lower energy consumption than the electrical one in the same device. As an example, we reported a ZnO/PbS heterostructure artificial synapse, in which the energy consumption is 80 pJ in electrical modulation while only 4 pJ in light modulation [37]. Recent studies distinguish three types of the photon synapses: photo-electric mixed, single optical and fully optical.

**Figure 5.**
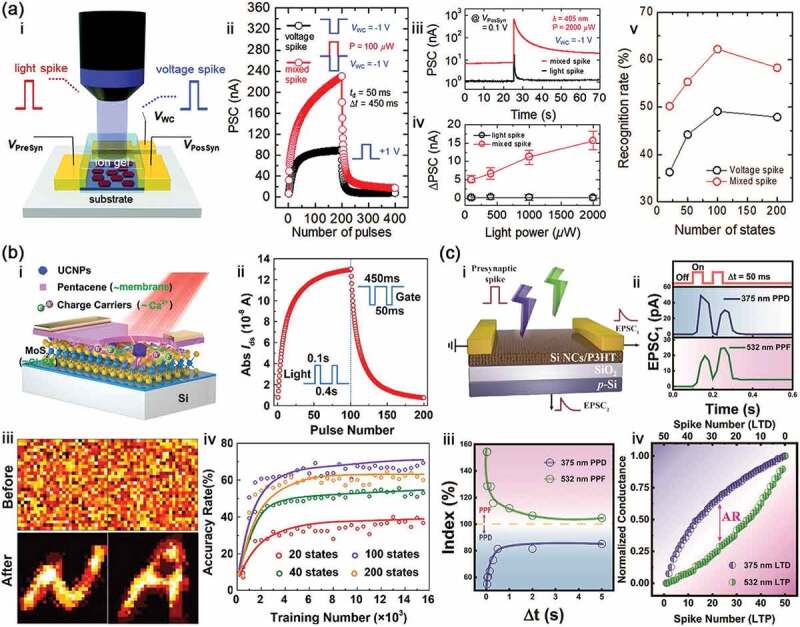
Light synapses and their characteristic. (a) Artificial synapse of photo-electric mixed type: i, schematic illustration of the photo-electric synapse; ii, LTP/LTD characteristics triggered by consisting of photo-electric mixed (red) and electrical (black) spikes; iii, EPSC responses induced by these spikes; iv, extracted ΔEPSC responses triggered by these spikes as a function of light power; v, recognition rates for photo-electric mixed and electrical modes as a function of number of weight states. Reproduced with permission from reference [67]. Copyright 2018 Wiley-VCH. (b) Artificial synapse of single light type: i, schematic illustration of phototransistor synapse device; ii, LTP/LTD characteristics triggered by light pulses (potentiation) and voltage pulses (Depression); iii, mapping images of letter ‘N’ and ‘A’ obtained before and after 15,000 training; iv, the accuracy rates for the case at different weight states. Reproduced with permission from reference [68]. Copyright 2020 Elsevier. (c) Artificial synapse of fully light type: i, schematic illustration of fully light synapse device; ii, EPSC1 induced by two consecutive optical spikes of 375 nm and 532 nm light; iii, dependence of the PPD and PPF index on δt; iv, LTP/LTD characteristics triggered by 532 nm light pulses (Potentiation) and 375 nm light pulses (Depression). Reproduced with permission from reference [69]. Copyright 2021 Wiley-VCH.

The photon synapses of photo-electric mixed type are based on the electrical synapses and combine with continuous illumination or light pulses to improve the synaptic plasticity. Sun et al. [67] have reported this mixed-type photon synapse, wherein graphene oxide (GO) nanosheets modified with long alkyl chains are embedded as a charge-trapping layer between the ion-gel dielectric and the indium-gallium-zinc oxide (IGZO) semiconductor (Figure 5(a)). In this device, several important forms of synaptic plasticity, including EPSC, IPSC, LTP and LTD, are successfully achieved in the pure electric mode. More importantly, when the light is introduced, these forms are improved so that the amplitude change of PSC (∆PSC) becomes much greater than the one for only electrical pulses with the same number of pulses and is in direct proportion to the light power. PSC in this mixed mode is three times larger than in the electrical mode after 200 pulses, which results in a much higher recognition rate when using these PSCs in the pattern recognition. However, in the photo-electric mixed type, light is only an auxiliary approach, and strictly speaking, this mode is not a real optical synapse. In contrast, single optical and fully optical artificial synapses display the importance of light because the light can be used to simulated these forms of synaptic plasticity.

The photon synapses of single light type are that light is used to realize EPSC or IPSC in the artificial synapses. Due to photoelectric effect, the number of carriers can be increased by absorbing photons in the artificial synapses. Therefore, light is used to mimic the EPSC while electricity is to simulate IPSC in this type photon synapses. In general, ultraviolet (UV) light with higher energy can be absorbed easily to generate more carriers, so it is often used to mimic excitatory synapses [70–75]. For example, Han et al. reported a series of materials for UV (365 nm) excitatory synapses, such as CsPbBr_3_ quantum dots (QDs) [70], MXene-ZnO [71] and hybrid carbon dots/silk protein [72]. Kim et al. [73] used indium-gallium-zinc-oxide (IGZO) to prepare an excitatory synapse under 380 nm UV-light. In the human visual system, light signals with different wavelengths are detected and processed by the retina, and are transmitted via the optic nerve to the brain for processing and memory [76]. More importantly, we can perceive the colorful world outside through our visual nervous system based on the response to different wavelengths of light. For photon synapses, it is very necessary that different wavelengths of light are used to simulate the plasticity. For example, Jo et al. [77] proposed an artificial photonic synapse by employing the size-mixed quantum dots (QDs) with three different optical bandgaps representing red, green and blue. The excitatory synaptic features, such as PPF, STP and LTP, have been activated in this device under the three visible lights with the wavelength of 405 nm (blue), 519 nm (green) and 635 nm (red). Similarly, Hong et al. [78] also used these three colors of light (blue: 457 nm, green: 532 nm and red: 660 nm) to mimic the excitatory synaptic plasticity. Furthermore, near-infrared (NIR) synapses can offer a remote-control approach to implement neuromorphic computing for data safety (information encryption to prevent them leakage) and artificial retinal system applications [79,80]. As such, Zhai et al. [68] developed a NIR synaptic device based on upconverting nanoparticles (UCNPs)-MoS_2_ as floating gate in the phototransistor, in which MoS_2_ acts as light-sensitive ion channels to reabsorb the visible light emitted from UCNPs under NIR illumination (Figure 5(b)). As a result, LTP multiple conduction state is performed under pulses (λ_NIR_ = 980 nm, P_light_ = 0.518 mW/cm^2^, t_d_ = 100 ms, Δt = 400 ms), and corresponding LTD state is obtained from the electrical pulses of gate (Vg = −40 V, t_d_ = 50 ms, Δt = 450 ms). On the basis of experimentally measured LTP/D characteristics of these NIR synapse for pulse state ranging from 20 to 200, ANN is simulated to realize pattern recognition for Modified National Institute of Standards and Technology (MNIST) and notMNIST with the higher recognition accuracy rate at 200 pulses state.

Through the photon synapses have been achieved by utilizing a compulsory combination of electrical and optical signals, while the problem of high-power consumption is remained. The fully light tunable synapses can achieve the ultralow power consumption, and also render ultrafast processing speed due to high bandwidth and low parasitic crosstalk [81]. For these reasons, lots of fully light synapses are developed in recent years [69,81–85]. As shown in Figure 5(c), Pi et al. [69] prepared a photon synaptic device based on the hybrid structure of silicon nanocrystals (Si NCs) and poly(3-hexylthiophene) (P3HT), which can work as the three-terminal transistor that has wavelength-selective synaptic plasticity and that the two-terminal device that mimics SRDP and metaplasticity with optical stimulation. More importantly, the excitatory (PPF and LTP) and inhibitory (PPD and LTD) features are achieved in the two-terminal device by two consecutive optical spikes with the interval (Δt) under the 375 nm illumination and 532 nm illumination. Liu et al. [82] developed a plasmonic photon synapse by relying on the effects of localized surface plasmon resonance (LSPR) and optical excitation in an Ag-TiO_2_ nanocomposite film, and realized the fully light-induced synaptic plasticity under visible and ultraviolet light stimulations for potentiation and depression, respectively. Meanwhile, we also demonstrated the fully light synapse by using ZnO/PbS heterostructure (Figure 2(c)), in which potentiation and depression are mimicked by UV (365 nm) and NIR (980 nm) light, respectively. In the fully light modulation, the recognition rates for notMNIST letter patterns have reached to 67 ± 6% being compared to that of electrical mode (68 ± 6%) [37].

## 4. Artificial neurons

Unlike the synapses, neurons have more complex structures and more functions to process and store the information. In electrical devices, artificial neuron is often composed of two or more artificial synapses, or integrated by artificial synapse with other functional devices. Similar to biological system, artificial neurons also work on information integration and memory, such as spatiotemporal information processing, cluster analysis and sensory memory. In this part, we will introduce this way for information processing and sensory memory.

### 4.1. Information processing

From part 2, we can know that a neuron can connect with thousands of pre-neurons through dendrites via synapses. In other words, one neuron receives information from other thousands of neurons. Therefore, it is of great importance to the neuron system that the information is filtered and integrated during the process of its propagation in neuron.

#### 4.1.1. Integrate and fire

**Figure 6.**
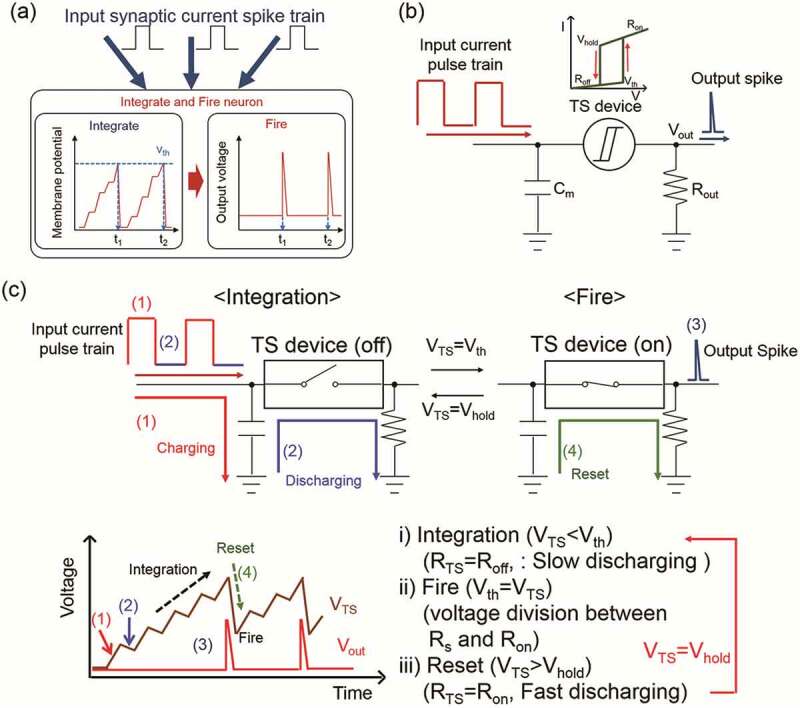
(a) Concept of I&F artificial neuron. (b) Schematic illustration of the TS-based I&F artificial neuron circuit. (c) Operation principle of TS-based I&F artificial neuron. Reproduced with permission from reference [95]. Copyright 2019 Wiley-VCH.

Some models can be proposed to explain how a neuron works, such as Hodgkin-Huxley (HH) model [86,87], McCulloch-Pitts (MP) model [88,89], oscillation model [90,91] and integrate and fire (I&F) model [92,93]. Among them, I&F concentrates on whether a neuron should fire a spike as action potential to be transmitted to the next neuron or not by comparing the local graded potential (LGP) with the threshold. Two forms of I&F have presented in neuron: integrate-and-fire (IF) and leaky integrate-and-fire (LIF). Therein, IF neuron will retain LGP boosting forever until it fires even when it receives a subthreshold signal, whereas LIF neuron leaks out LGP in a short time when it is lower than the threshold [94]. I&F model can be well described in Figure 6(a). Simply, the input current is integrated and the membrane potential is charged in the I&F neuron. Once the membrane potential reaches the threshold voltage (V_th_), the neuron will generate spikes to the next neuron and reset the membrane potential. Usually, the threshold switching (TS) devices, including resistive random access memory (RRAM) [96], flash memory [97], phase change random access memory (PRAM) [98], ferroelectric thin-film transistors and electrolyte film transistors [11], are used to mimic the I&F neuron, and also have to be combined with other types of devices such as capacitor and resistor, in which a circuit can be well employed to implement this function (Figure 6(b)). In this, the capacitor is as the membrane capacitor (C_m_) to integrate the input current, and the resistor represents an output resistor (R_out_) to generate output spikes by voltage division with the TS device. The input electrical pulses and their interval charge and discharged the capacitor (Figure 6(c)), respectively. When the TS device is initially in the off-state, charging effect is over discharging one due to the slow discharge. In this case, the total charges increase as the pulse is applied. Next, once the increased charges make the voltage drop of the TS device (V_TS_) reach V_th_, the neuron circuit starts the fire process and generates the output spikes (V_out_) because the TS device switches from the off-state to the on-state. At the same time, the capacitor is discharged quickly so that V_TS_ decreases and the TS device returns to the off-state (reset). Then, this neuron circuit returns to the integration process [95].

**Figure 7.**
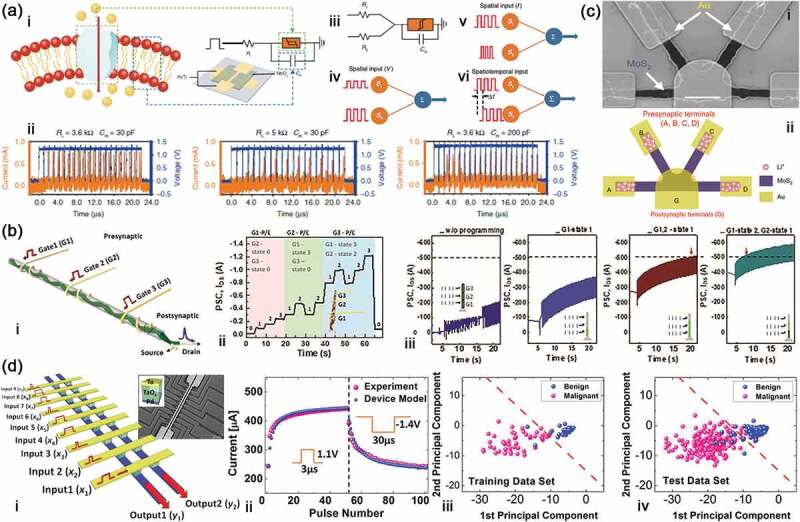
Information processing in artificial neurons. (a) Information processing of I&F and neuronal integration in the artificial neuron: i, schematic illustration of an ion channel embedded in cell membrane of a biological neuron with corresponding circuit based on NbO_x_ memristor for a spiking neuron, and inset is schematic diagram of this memristor; ii, characterization of the I&F neuron under a continuous pulse train with different capacitance (C_m_) and resistance (R_L_); iii, the circuit diagram of the spiking neuron; schematic illustration of spatial summation with different input pulse amplitudes (iv), different input pulse intervals (v) and different time intervals of input pulses (vi). Reproduced with permission from reference [99]. Copyright 2020 Nature. (b) Neuronal integration in dendritic network transistor: i, schematic illustration of the transistor; ii, reproducible and discrete channel conductance feature of the transistor; iii, information integration in the transistor. Reproduced with permission from Reference [103]. Copyright 2021 Wiley-VCH. (c) Neuronal integration in multiterminal 2D device: scanning electron micrographs (i) and schematic illustration (ii) of this device. Reproduced with permission from reference [104]. Copyright 2019 nature. (d) Feature extraction in memristor array: i, schematic illustration and scanning electron micrographs of the memristor array and relevant operation in the array device; ii, LTP/LTD characteristics in the memristor array through experiment and simulation; Classification based on the trained memristor network of training process (iii) and application (iv). Reproduced with permission from reference [105]. Copyright 2017 American Chemical Society.

Nowadays, researchers are paying more attention to I&F function of neuron, and lots of devices are used to mimic successfully it [95,99–102]. For example, Hwang et al. [95] employed three different types of TS devices: NbO_2_-based insulator-to-metal transition (IMT) device, B-Te-based ovonic threshold switching device and Ag/HfO_2_-based atomic-switching TS device to investigate the effect of the switching parameters of TS devices and the characteristics of TS-based I&F neurons through the above circuit of Figure 6. Therein, they confirmed that the off-state resistance of the TS device determines the leaky/nonleaky characteristic of the I&F neuron, and the switching speed decides the types of the activation function of neuron (ReLU & Sigmoid). Meanwhile, they also found that the TS-based I&F neuron devices have promise for neuromorphic pattern recognition system applications due to the small device area, low power operation power (≈fJ per spike), and low process temperature (<400°C). Yang et al. [99] used a simple circuit including a volatile memristor, a parallel capacitor (C_m_) and a load resistor (R_L_) to implement the I&F neuron as displayed in Figure 7(a-i,ii). Herein, the memristor is Pt/Ti/NbO_x_/Pt/Ti that mimics the dynamics of an ion channel located near the soma of a neuron, while the parallel capacitor represents the capacitance of cell membrane. In the circuit, once the voltage applied to the capacitor reaches V_th_, the NbO_x_ memristor will switch to on-state from off-one and the artificial neuron will be fire. After firing, the voltage can across the load resistor and the memristor will be redistributed, so that the capacitor begins to discharge. As result, the memristor returns to off state after the voltage drops below V_hold_. This change is in accord with the above description in the Figure 6. In particular, the characteristic of the I&F neuron strongly depends on R_L_ and C_m_, in which the small C_m_ or low R_L_ can accelerate the fire process. Meanwhile, they have also demonstrated that this characteristic can also be regulated by adjusting the pulse parameters, such as the spiking frequency and voltage.

#### 4.1.2. Neuronal integration

In the nervous system, when the stimuli signal is transmitted in the neurons, the continuous and variable action potential are integrated as an excitatory or inhibitory information to the postsynaptic neuron from different presynaptic neurons. The neuronal integration is an important step of this transformation, which mainly includes spatial summation and temporal summation [106,107]. Spatial summation involves events that occur simultaneously in different synapses, while temporal summation is taken over non-simultaneous events. Spatial and temporal summation can coexist in one neuron to display the spatiotemporal information processing in the nervous system. Therefore, it is a vital significance for computation and memory in neuromorphic hardware to emulate this function using the artificial neurons. In order to emulate this function, an additional resistor was added into the circuit of I&F neuron in Figure 7(a-i) to form the new circuit in Figure 7(a-iii). The spatial integration has been observed by applied simultaneously stimulus on the two load resistors, and also relies on the pulse voltage and frequency (Figure 7(a-iv,v)). Interestingly, the firing frequency of the artificial neuron is a function of the time interval (∆t) between the two pulse trains, in which different time intervals generate different firing states (Figure 7(a-vi)) [99].

In view of biological synaptic communication mechanism and dendritic structure of a neuron, an artificial neuron should be multichannel communication that can regulate the synaptic-weight and integrate the spatiotemporal signal [11]. Recently, Lim et al. [103] proposed an organic transistor with the dendritic network architecture, consisting of a double-stranded assembly of electrode microfibers, an ion-gel gate insulator and carboxylic-acid-functionalized polythiophene as semiconductor channel (Figure 7(b)). In the unit neural network, the synaptic weight is represented by the multigate microfibers, and can be controlled independently by the corresponding gate to process the spatiotemporal signals. Therein, the levels of synaptic weight can be controlled and reproduced back and forth by one gate at a time even though other synaptic weights are programmed at a specific level. Meanwhile, they have also demonstrated that the integrated response spikes can be classified according to the combination of the programmed state levels and the spatiotemporal signals from the specific gates by utilizing the characteristics of the multigate for the same input. Furthermore, Lu et al. [104] used a two-dimensional material of MoS_2_ modified by Li+ ions to build a multiterminal neuromorphic transistor, in which the conductivity of MoS_2_ can be adjusted by controlling the migration of Li+ ions with an out electric field (Figure 7(c)). In this device, one MoS_2_-memristor is deemed to be one synapse, and the synaptic competition and synaptic cooperation effects for signal integration are simulated in this simple bio-inspired artificial neural network.

In recent years, neuronal integration is getting more and more attention in memristor and neuromorphic device [108–112]. For example, Huang et al. [108] adopted metalloporphyrin/oxide hybrid heterojunction to develop a novel multifunctional memristor, and the signal filtering function of the biological visual system was emulated in these memristive arrays. Shang et al. [109] reported a memristive array based on Nb_2_O_5_ and Li_x_SiO_2_ as the channel and electrolyte gate materials, and utilized the spatiotemporal information processing to detect moving orientation in a tactile sensing system. Zhou et al. [110] designed a memtransistor based on MoS_2_/WSe_2_ van der Waals heterostructure, which exhibited significant potential for high-order spatiotemporal recognition.

#### 4.1.3. Feature extraction

Feature extraction is a way for information integration, selection and classification, which aims to reduce the dimensionality of the data by transforming the original input data into a new space based on identified feature and is widely applied to machine learning and pattern recognition [105,113,114]. Memristors, as a promising candidate for neuromorphic computing systems, are very suitable to implement the feature extraction by constituting the structure of crossbar array, because the vector-matrix multiplications and conductance (weight) updating are easily carried out in the memristor crossbar arrays [115,116]. For this reason, Choi et al. [105] fabricated a small memristor crossbar array with 9 × 2 memristors based on Ta_2_O_5_ material to perform the feature extraction (Figure 7(d-i)). The Ta_2_O_5_ based memristor has good resistive switching characteristic, and displays excitatory and inhibitory conductance under voltage pulses (Figure 7(d-ii)). In the memristor crossbar, the rows and columns are considered as the input and output channels, respectively. The output vectors are determined by the vector-matrix dot-product of the input signal and the memristor weight matrix after inputting the voltage pulses with different pulse widths that represent the standard breast cancer data set from the University of Wisconsin Hospital. By this way, combining the principal component analysis (PCA), the cells are classified as malignant or benign automatically through the labelled data training (Figure 7(d-iii,iv)). The result is that only 17 data points among 583 test data ones are misclassified, which reaches to 97.1% accuracy close to results obtained by directly solving the eigenvectors in software (97.6%). The superiority for this memristor crossbar is that it greatly simplifies or avoids complex calculations that consume a large amount of hardware resources.

### 4.2. Sensory-memory neurons

Before memory and learning, the brain receives external stimuli from every sensory system in the body, such as visual, auditory, olfactory, gustatory, tactile and pain nervous systems [117,118]. When the stimuli are transmitted into brain, the synaptic activity is activated to learn and memory the external information and things. This function can be realized in electrical devices, called sensory memory, which can enable the awareness of motion for various electronic products. In this part, we will introduce these sensory memory devices, including retina, tactile, auditory, olfactory and nociceptive memory, and show how they simulate synaptic plasticity in response to external stimuli from the sensory system.

#### 4.2.1. Artificial retina neurons

**Figure 8.**
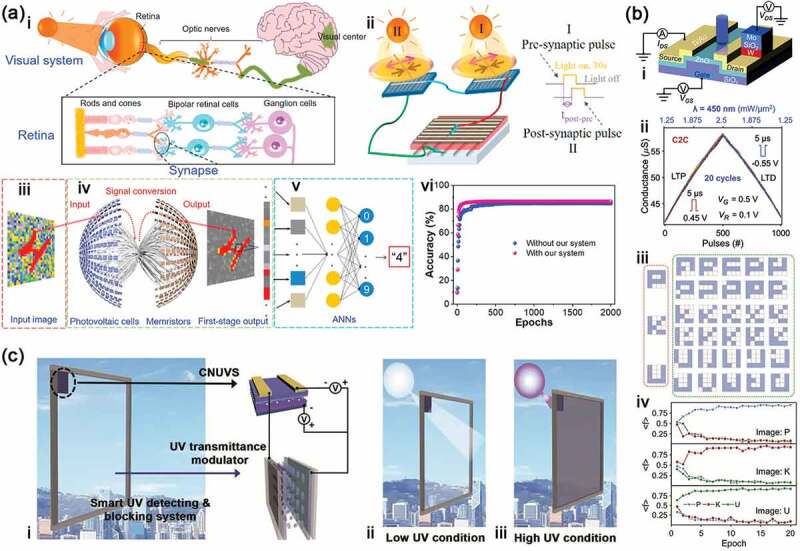
Artificial retina neuron systems. (a) Self-powered artificial retina perception system: schematic illustration of biological (i) and self-powered artificial (ii) visual system; iii and iv, illustration of the image preprocessing based on self-powered artificial retina perception system; v, illustration of ANN for image recognition; vi, image recognition rate with and without the image preprocessing. Reproduced with permission from reference [122]. Copyright 2020 Elsevier. (b) Artificial retina neuron systems based on one-phototransistor-one-memristor array: i, schematic illustration of the device structure; ii, near-linear LTP/LTD characteristics triggered by positive and negative voltage pulses; iii, training and test set of the letters P, K, and U; iv, activation function output with three classifications in 20 training epochs. Reproduced with permission from reference [123]. Copyright 2022 Wiley-VCH. (c) Smart system to detect and block UV: i, schematic illustration of the system; the smart system for UV detecting (ii) and blocking (iii). Reproduced with permission from reference [124]. Copyright 2020 Wiley-VCH.

As discussed in part 3.2, most of external information is captured by human retina into our brain. Therein, it takes up near half of our cerebral cortex to process and integrate the information, so that we can appreciate an object including its size, shape, color, brightness, distance, location, smoothness, roughness [119,120]. In artificial neuron, the retina memory often has two parts: photoelectric device and memristor. The light is converted into electrical signal through photoelectric device to arouse the neural plasticity in memristor. These devices can constitute the artificial neural networks to finish the task of pattern recognition like optic nerve system through a series of processes including image pre-processing, data reduction, segmentation, object recognition and image understanding [121]. For example, Yang et al. [122] presented an artificial retina neuron system for pattern recognition and visual function emulation by employing perovskite-based memristors as artificial synapses and polycrystalline silicon solar cells as the artificial retina (Figure 8(a)). The perovskite-based memristors with a structure of ITO/CsPbBr_2_I/poly(3-hexylthiophene)(P3HT)/Ag show multiple types of synaptic plasticity, such as STP, LTP, PPF, SRDP and STDP. By a light stimulation with various wavelengths and intensities, the electrical output signal is generated in the solar cell and subsequently transferred to the memristor. On the one hand, the electrical signal connects information from the outside visual world due to the broadband light response of solar cell from visible to near-infrared light. On the other hand, it is also acted as an energy source to operate the memristor so that the artificial retina neuron system could be self-powered. Especially, the retina perception system has the ability of feature extraction and classification to realize some functions of convolutional neural networks (CNNs) with improved recognition rate, boosted recognition speed, and reduced energy consumption. In particular, the accuracy for pattern recognition reaches to 85.45% with the artificial retina neuron system but only 77.31% without it.

In addition, other lots of articles have also reported this artificial retina neuron systems made from various memristors or neuromorphic devices, which not only improves some performances of these devices but also give them novel functions [123–127]. Huang et al. [123] reported a system with the Mo/SiO_2_/W memristor and the ZnO phototransistor to construct one-phototransistor-one-memristor (1PT1R) photon neuron system (Figure 8(b)). This 1PT1R device exhibits highly linear weight updates according to light programming and highly uniform multilevel conductance states without sneak path problems. Meanwhile, the ANN system with 1PT1R memristor array has fast and highly efficient training in the experiment and yields an image recognition accuracy of 99.3% after the training process, due to the excellent performance of the 1PT1R array, especially linear switch to a specific conductance state. Lee et al. [124] developed a smart system that can detect and block UV light by integrating C_3_N_4_ photonic synapse and UV-transmittance modulator (Figure 8(c)). Therein, the synaptic weight of C_3_N_4_ photonic synapse can be changed by the conditions of UV exposure. When the excess UV rays irradiate the system, the synapse device can regulate the UV-transmittance modulator to make sure the right UV light pass through, which is helpful to develop the advanced electronic skin and smart glasses to protect our health.

#### 4.2.2. Artificial tactile neurons

**Figure 9.**
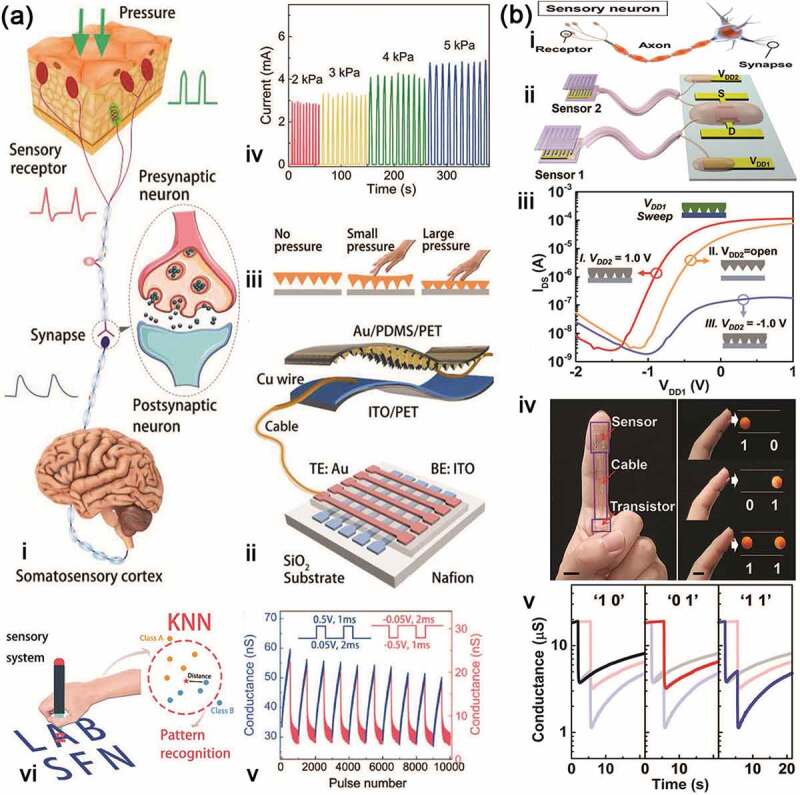
Artificial tactile neuron systems. (a) Bioinspired artificial sensory nerve based on Nafion memristor: schematic illustration of the biological tactile perception system (i) and artificial tactile perception system (ii); iii, illustration of the sensor under zero, small and large pressure states; iv, current responses of pressure sensor under different pressure pulses; v, LTP/LTD characteristics of nafion memristor; vi, applied at the writing of English characters by the sensory nerve system assembled on a ‘pen’. Reproduced with permission from reference [128]. Copyright 2019 Wiley-VCH. (b) Artificial sensory neuron with tactile perceptual learning: comparison of the biological sensory neuron (i) and artificial NeuTap (ii); iii, transfer curves of the transistor under the modulation effect of sensor; iv, digital image showing the NeuTap; v, typical responses of the NeuTap to three types of pattern pairs from iv. Reproduced with permission from reference [129]. Copyright 2018 Wiley-VCH.

Tactile neuron is also an important part of our sensory system, which can help us interact with the external environment depending on the comprehensive activities of touch sensing, refining and learning. For example, when we want to hold a fragile object, a few failures will tell us how much force should be applied. Physiologically, a biological tactile neuron has three key components: sensory receptor cell, neural passage, and sensory perception part in the brain (Figure 9(a-i)). In this tactile system, the receptor is on sensory neuron embedded in the skin. Once it detects the touch signal, it will send the signal to synapse through a long chain of afferent axons, and to the next neurons for further process. Therefore, two functional components are necessary to develop the artificial tactile neuron: pressure/touch sensor that translates external physical stimuli into voltage pulses, and artificial synapse that integrates and converts these voltage pulses into postsynaptic current. For this reason, Zhang et al. [128] designed an artificial haptic neuron system with a piezoresistive pressure sensor as a sensory receptor to transform mechanical stimuli into electric signals and a Nafion-based memristor as the synapse to further process the information (Figure 9(a-ii)). The sensor is two-electrode configuration: the top electrode (TE) is the active layer with a micropyramid structured Au/polydimethylsiloxane (PDMS) film attached on a polyethylene terephthalate (PET) substrate, and the bottom electrode (BE) is indium-tin-oxide supported ITO/PET. When forcing certain external pressure, the spires of these Au pyramids will contact with the BE and form lots of conductive connections, so that the output current will be abruptly increased (Figure (9a-iii,iv)). The artificial synapse is a crossbar-structured memristor based on Au/Nafion/ITO, displaying excitatory and inhibitory characteristic (Figure 9(a-v)) that can simulate well some synapse function such as PPF, PPD and STDP. These two devices are connected by Cu wire to constitute the artificial haptic neuron system, which can detect tactile stimuli encoded with temporal information. Therein, the system is employed to identify English characters ‘L’, ‘A’, ‘B’, ‘S’, ‘F’ and ‘N’ through a smart pen (Figure 9(a-vi)) with a high accuracy of 91.7%.

Compared with artificial retina neurons, the artificial tactile memory system has another characteristic of flexibility, which helps to develop skin-electronics and humanoid robots [130,131]. The tactile neuron system can give these apparatuses flexibility and intelligence when coming to even familiar objects. As discussed above, this system often integrates sensing and memory elements to accept and process external stimuli. Chen et al. [129] reported a neuromorphic tactile processing (NeuTap) system that simulates the sensory neuron and shows the function of perceptual learning (Figure 9(b)). In their design, a resistive pressure sensor, a soft ionic cable and a transistor represent the receptor, axon and synapse to sense, transmit and process the information, respectively. Similarly, the resistive pressure sensor also uses this pyramidal structure based PDMS coated with carbon nanotubes (CNTs), and displays good pressure sensing characteristics. As a proof-of-concept, they also used the NeuTap system to implement the tactile pattern recognition (Figure 9(b-iv,v)). In the recognition process, the NeuTap system was attached to a finger, and was defined as ‘1’ and ‘0’ along with convex and flat changes of the finger, respectively. The result shows that the ‘11’ pattern has the largest conductance change due to the two successive pressure stimuli, while the conductance response to ‘01’ pattern is a little higher than to ‘10’ pattern after move-touch action because the response to the ‘10’ pattern decays earlier than that of the ‘01’ pattern. By this way, the signal processing for the pressure spatiotemporal information is carried out in the artificial tactile neurons. In addition, the self-powered tactile neuron is also developed by employing the triboelectric nanogenerator to produce pressure-triggered electric signals without the need for an external power supply [132].

#### 4.2.3. Artificial auditory neurons

**Figure 10.**
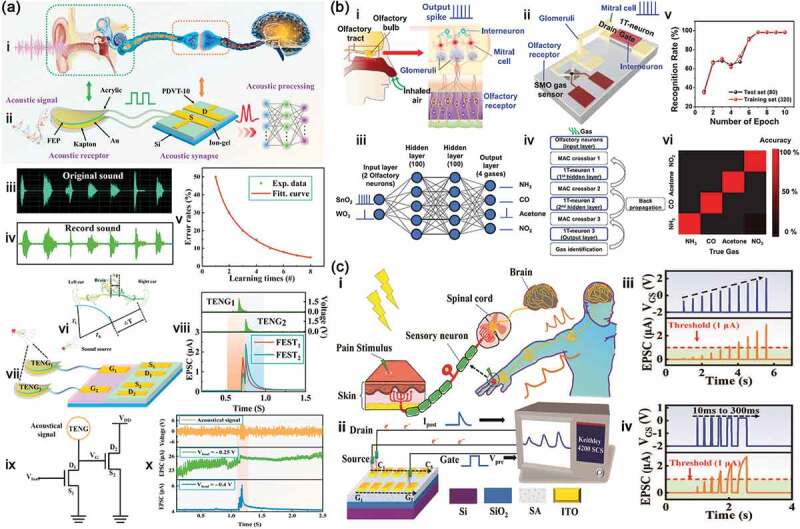
(a) Artificial auditory neuron: comparison of the biological auditory neuron (i) and a self-powered artificial auditory neuron (ii); original sound wave (iii) and record sound wave (iv) in this artificial auditory neuron; v, recognition error rates as a function of learning times; vi, illustration of sound location by binaural effect in the human brain; vii, illustration of sound location in two artificial auditory neurons; viii, postsynaptic current when the sound comes from the right direction; ix, illustration of the noise-adjustable neuromorphic circuit; x, output current of the neuromorphic circuit with different load voltage under noisy environment (100 dB). Reproduced with permission from reference [133]. Copyright 2020 Elsevier. (b) Artificial olfactory neuron: I, comparison of the biological olfactory (i) and an artificial olfactory neuron module composed of a SMO gas sensor and a MOSFET-based 1T-neuron (ii); iii, illustration of multi-layer spiking neural network (SNN) constructed for gas classification; iv, flow chart of the simulation for the gas classification; vi, the accuracy for gas classification of four gases. Reproduced with permission from reference [134]. Copyright 2022 the Authors. (c) Artificial nociceptive neuron: comparison of the biological pain-perception process (i) and artificial nociceptive neuron of the oxide transistor array connected to the test system (ii); EPSC responses triggered by voltage spikes with different pulse amplitude (iii) and pulse durations (iv). Reproduced with permission from reference [135]. Copyright 2022 the Royal Society of Chemistry.

As with retina and tactile neurons, auditory neuron is also one of the most important and efficient sensory system for our human beings that can detect, process and store the acoustic signal. In the auditory pathway (Figure 10(a-i)), auricle collects the external acoustic signal and then causes the eardrum to vibrate. After amplified by the ossicular chain, the vibrate is transmitted to the inner ear. When sound or vibration reaches the cochlea, it is converted into electrical signal by the hair cells. After that, the electrical signal is transferred to the neural center that integrates, analyses and stores the massive information [136,137]. The pathway for the acoustic signal in biological system will inspire us to exploit the artificial auditory neurons. For example, Wan et al. [112] reported a series of capacitively coupled multiterminal neuro-transistors based on the proton-conducting solid-state electrolyte film to realize spatiotemporal information processing by mimicking the dendritic discriminability of different spatiotemporal input sequences. Resulting from this processing, sound location functionality of the human brain was also emulated on the multiterminal neuro-transistors. Wu et al. [138] developed a neural network architecture based on HfO_x_ memristor array with the function of handling complete sound signals received by two artificial ears.

Like most artificial sensory neurons, artificial auditory neuron usually has two parts: sensor and memory device, which can integrate sensing and storing functions for a more efficient artificial auditory system. Inspired by the biological auditory self-adaptation, Chen et al. [133] fabricated a self-powered artificial auditory neuron by triboelectric nanogenerator (TENG) and a field effect synaptic transistor (FEST) to emulate the biological auditory functionalities, which also realized self-adaptation artificial auditory pathway by constructing an intelligent auditory neuromorphic circuit (Figure 10(a-ii–x)). TENG is functioned as acoustic receptor in response to voice in a broad frequency range, consisting of an Au covered annular acrylic sheet, a fluorinated ethylene propylene (FEP), an Au covered Kapton membrane and an annular acrylic sheet (Figure 10(a-ii)). The TENG device has high sensitivity (129 mV/dB) and can accurately record different sound wave signals (Figure 10(a-iii,iv)). FEST with an electric double layer (EDL) structure is fabricated using ion-gel as the gate dielectric layer to integrate, analyze and store the voice instructions (Figure 10(a-ii)), which can mimic some synaptic plasticity such as EPSC, PPF, STP and LTP. The artificial auditory system can distinguish some word instructions (B, C, E, G, sound level is 90 dB) with overall accuracy of 95% after 8 times training (Figure 10(a-v)). The sound location can also be emulated the auditory function of the left and right ears through combining these two systems (Figure 10(a-vi–viii)). Meanwhile, they have designed an intelligent auditory neuromorphic circuit to adjust the noise and realize the self-adaptation artificial auditory pathway, which provides practicability of the artificial auditory pathway at noisy environment (Figure 10(a-ix,x)).

#### 4.2.4. Artificial olfactory neurons

Nowadays, gas sensor is increasingly important in our life for gas monitoring, food quality and healthcare applications such as breath based early diagnosis of diseases [139–141]. With the development of artificial intelligence, more and more gas sensors are integrated with memristors/neuromorphic devices to simulate human olfactory system. Li et al. [142] used 2D covalent organic framework (COF) film to develop a gas artificial synapse that can identify the alcohol atmospheres. Inspired by camel noses, Huang et al. [143] developed a highly sensitive and ultradurable neuromorphic capacitive humidity sensor that exhibited a robust capability to discriminate moisture from other volatile compounds. In the biological olfactory sensing system (Figure 10(b-i)), when the gas is sucked up into the nose, the odorant stimulates the olfactory receptors so that the chemical reactions between them trigger electrical signals as an output. These electrical signals are then transmitted to the olfactory bulb through glomeruli. Mitral cells and interneurons in the olfactory bulb can preprocess and transmit the electrical signals into the brain olfactory cortex to identify the odor.

Inspired by the biological olfactory nervous system, Choi et al. [134] designed an artificial olfactory neuron to perform gas sensing and spike encoding by integrating a chemoresistive gas sensor and a single transistor neuron (1T-neuron), as shown in Figure 10(b-ii). In this artificial system, the gas sensor is considered as olfactory receptor in the biological system to detect odorants, which uses semiconductor metal oxide (SMO) as sensing material and displays variable resistance by adsorbing different gases. The 1T-neuron is a metal-oxide-semiconductor field-effect transistor (MOSFET) that can realize the neuronal I&F function. Between them, metal interconnection is used to transport signals from gas sensor SMO to 1T-neuron, corresponding to the glomeruli that transports signals from the olfactory receptors to the mitral cell. The artificial olfactory neuron can perform gas detection and spike encoding simultaneously, which shows the ON- and OFF-type responses and the inhibitory function for efficient odor classifications. Meanwhile, they performed the semiempirical simulations with Python software by using the measured electrical properties of the artificial olfactory neuron to classify four gases of NH_3_, CO, acetone and NO_2_ (Figure 10(b-iii–vi)). As a result, the artificial olfactory neuron is demonstrated with a high accuracy of 98.25% after seven epochs, and then these four different gas species are well classified after the training.

#### 4.2.5. Artificial nociceptive neurons

Retina, tactile, auditory and olfactory neurons can tell us most of the information about an object, such as its color, size, whether it is hard or soft, and what sound and smell it makes, but they cannot tell if it is harmful for us. Nociceptor is also an important sensory receptor in biological system that can recognize external harmful inputs and transmit pain signals to the central nervous system to avoid potential damage [144,145]. When our skin is hurt, the stimulus (e.g. mechanical stress or extreme temperature) can activate the pain nerve ending to initiate the pain electrical signals. The nociceptor receives these signals and compares their intensity with the threshold value to decide whether or not to generate the action potentials. If the signal intensity is higher than the threshold, an action potential is generated and travels along the spinal cord to the brain (Figure 10(c-i)). Conversely, the signal intensity below the threshold cannot produce the action potential [135,146]. Except from threshold feature, the nociceptor also has others, such as relaxation, allodynia and hyperalgesia, which are related to the duration and repetition rate of the external stimuli [147,148].

Recently, lots of electrical devices have mimicked the nociceptor system to develop the artificial nociceptive neurons, which concentrates on the skin nociceptors and retina nociceptors [135,149–155]. Jiang et al. [135] prepared a 5 × 5 array of ionotronic junctionless indium-tin oxide (ITO) transistors by using sodium alginate (SA) biopolymer electrolyte as the common neurotransmitter layer for ion transmission to realize a nociceptor network and establish extensive soft connections between individual devices through a common electrolyte (Figure 10(c-ii)). The pain threshold of biological nociceptor has been presented through adjusting pulse voltages from 1.3 V to 4 V and pulse durations from 10 ms to 300 ms (Figure 10(c-iii,iv)). In addition, other characteristics of nociceptor can also be realized well, such as memory of prior injury and spatiotemporal sensitization. Xu et al. [149] reported a two-terminal lateral-structured device based on MAPbBr_3_ single-crystalline thin platelets, which can feel the pain from extreme temperatures (heat and cold). Kim et al. [150] implemented an all-oxide-based, highly transparent optical nociceptive memristor by using a ZnO/ATO/FTO heterostructure, which shows optically trigged nociceptive function with all five nociceptive features of ‘threshold’, ‘relaxation’ along with ‘allodynia’ and ‘hyperalgesia’, depending on the strength, duration, and repetition rate of the input.

## 5. Behavior simulation

The brain can make a well-reasoned decision based on the external stimuli and information processing in neurons. Behavior is the result of this brain function, such as conditioned reflex, instincts and habits of living things. In this part, we will introduce some classical behaviors that are mimicked in the memristors and neuromorphic devices.

### 5.1. Classical conditioning

Classical conditioning is a key way in the learning and the adaptability processes of the brain, which can help the body to get ready for an expected or likely event [156]. There are four characteristics of classical conditioning: acquisition, extinction, recovery and generalization, relating to storage of information, elimination of old information, re-memorization and storage of new information, respectively. The classical conditioning is demonstrated by Pavlov’s dog experiment. In the Pavlov’s dog experiment, the dog can salivate once watching the food, but the ring alone cannot cause the dog to salivate. If feeding the dog is accompanied with ringing the bell for a few training sequences, the dog can salivate when only hearing the bell. It has been demonstrated that the dog learned to associate the ring with food. After some time, the dog could forget so that the bell cannot arouse its saliva, while it will work again after a few tries. The whole experiment represents the process of learn, remember, forget and recall in the brain.

**Figure 11.**
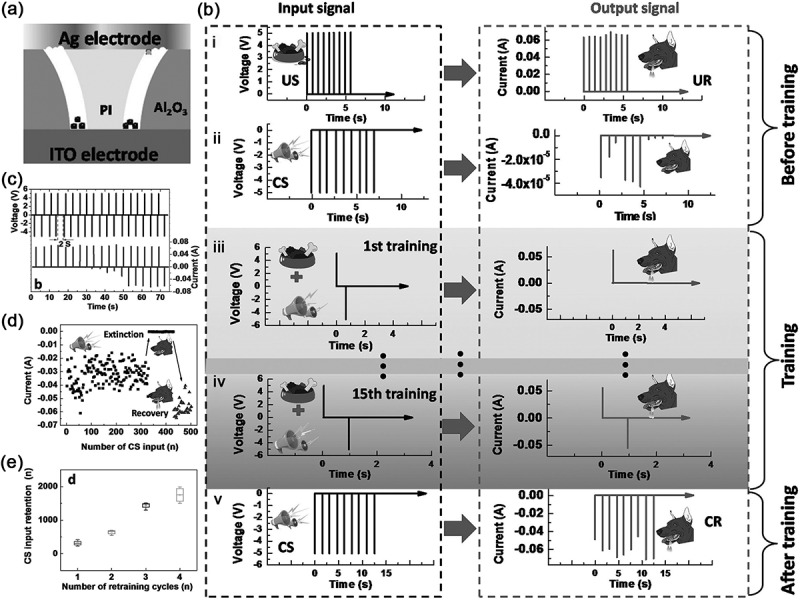
Mimicking classical conditioning of Pavlov’s dog experiments based on a single flexible memristor. (a) Schematic diagram of the single flexible memristor. (b) the process of the Pavlov’s dog experiment in the single memristor by positive and negative voltage pulses. (c) Pulse responses under alternating positive and negative pulse stimulations. (d) extinction and recovery of classical conditioning. (e) CS input retention recorded after different numbers of retraining cycles. Reproduced with permission from reference [157]. Copyright 2017 Wiley-VCH.

For this function, various memristors and neuromorphic devices are developed to simulate the Pavlov’s dog experiment [157–164]. In order to mimic this behavior more comprehensively, Yang et al. [157] utilized Al_2_O_3_ nanoparticle (NP) array:polyimide (PI) to design a simple two-terminal memristor with the structure of ITO/Al_2_O_3_ NP:PI/Ag (Figure 11(a)). In this device, Ag filaments can be formed easily in the Al_2_O_3_:PI stacking layer due to electrochemical metallization, and also can go through the processes of dissolution, re-deposition and thickening under positive and negative voltage pulses. During simulation process, positive voltage pulse is an unconditioned stimulus (US) that represents the food. In this case, the corresponding unconditioned response (UR) is the output current, displaying high-level value that can be as the salivation. The conditioned stimulus (CS) of the bell is represented by negative voltage pulse, which arouses the conditioned response (CR) when the output current reaches the threshold. Before training (Figure 11(b-i,ii)), only positive voltage causes the output signa over the threshold (salivation response). In the training process, the positive and negative voltage pulses are alternately applied to the device (Figure 11(b-iii,iv,c)). To 15th training, the negative voltage pulse has generated the high-level output current. After this sufficient training (Figure 11(b-v)), negative voltage pulses alone can also produce the high current output that is regarded as salivation response. This process is equivalent to acquisition in the brain that acquires get relevant information and memorizes it under a certain condition. However, the dog will forget the bell ringing and not salivate, while a little stimulation can quickly restore its response to the bell. Similarly, in this memristor, conditioned reflex will be extinguished under applied negative voltage pulses alone many times, and the conditioned response will be recovered after retraining (Figure 11(d)). Meanwhile, more retraining cycles will enhance the input retention, hinting the information will be more deeply remember and more difficult to forget, which is related with the dog behavior that the training of bell stimuli is repeated to achieve long-term memory about the food. In addition, Park et al. [158] developed a metal-chalcogenide (MC)/metal-oxide (MO) heterogeneous photonic neuro-transistor that can be used to mimic the Pavlov’s dog experiment. During the process, the UV and green light spikes are served as US and CS to represent the food and the bell ringing respectively, and the firing output current represents the salivation. By this way, the association conditioning and extinction process can be realized in this photonic neuro-transistor, which equals to simulate learning/memory of trained experiences and forgetting process of old information in human. Jiang et al. [164] reported a photoelectrically mixed phototransistor for emulating photoelectric-synergistically classical Pavlovian conditioning based on CsPbBr_3_-quantum-dots/2D-MoS_2_ heterojunction channel. Therein, light pulses can generate larger current than electrical pulses. Electrical is used as CS of the bell and light is as US of the food, in which the Pavlov’s dog experiment can be achieved in this photoelectrically mixed.

### 5.2. Protection awareness

**Figure 12.**
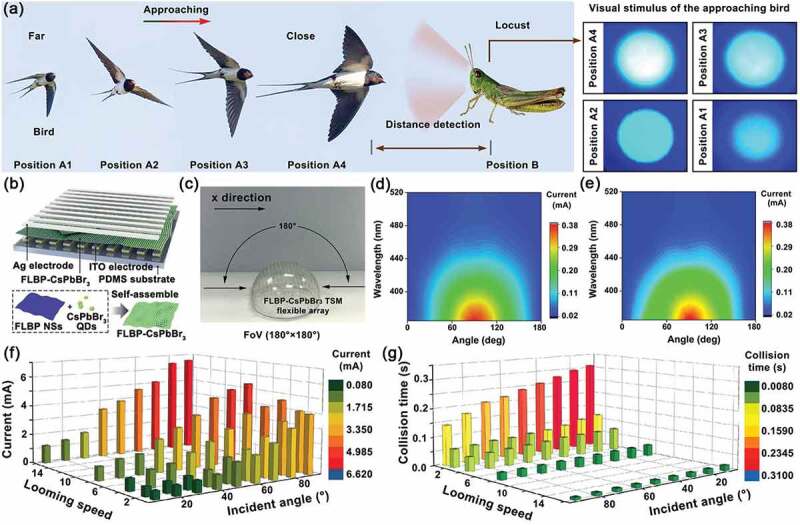
Mimicking biological avoidance behavior by using memristor. (a) illustration of approaching bird to the locust and snapshots of the stimulated visual information detected by the locust in the form of the monotonic increase in the optic power from position A1 to position A4. (b) Illustration of memristor structure. (c) Schematic of field-of-view of the biomimetic compound eye. Recorded respective device conductance with the different incident light along x direction (d) and y direction (e). (f) Inflection point values in the output current of the memristor for different looming object speeds or the different positioned device with specific incident angles. (g) Detected collision time before impact by the memristor for different looming object speeds or the different positioned devices with specific incident angles. Reproduced with permission from reference [167]. Copyright 2021 Nature.

In natural world, many living creatures have an instinct or protection awareness to avoid predators and obstacles, in which most of them depend on the visual stimulus to determine the distance of predators and obstacles from themselves. When their eyes observe the predator or obstacle, animals can anticipate collision so that the firing rate of neuron will change: increase, peak, and then decrease. The firing peak appears before the image reaches the maximum size in their eyes throughout the predator or obstacle approaching [165,166]. For this reason, Wang et al. [167] reported an artificial lobula giant movement detector (LGMD) visual neuron to mimic the escape behavior of locust from bird (Figure 12). The artificial neuron is implemented using 20 × 20 threshold switching memristor arrays with a single device structure of Ag/few-layer black phosphorus (FLBP)-CsPbBr_3_ heterostructure/ITO to mimic the compound eye of locust (Figure 12(b,c)). Due to the hemispherical shape of this biomimetic compound eye, the identical incident angle of 180° along both the x and y direction is viewed as the field-of-view. The strongest photocurrent output is obtained when the light is incident at 90°. Meanwhile, the hemispherical compound eye exhibits wavelength discrimination in the UV–visible range (Figure 12(d,e)). Due to this property, the artificial neuron can be used to evaluate the position of the approaching object and the collision time (Figure 12(f,g)), which is like a locust perceiving a bird coming to prey. Similarly, Kim et al. [168] present an artificially intelligent magnetoreceptive synapse based on a ferroelectric-polymer-gated field-effect transistor with an air-suspended gate electrode laminated with an elastic polymer composite containing superparamagnetic particles. This artificial synapse facilitates sensing, memorizing, and learning of various magnetic fields, which can be used to mimic the barrier-adaptable navigation and mapping of a moving object of birds. The protection awareness to avoid predators and obstacles can be helpful to develop the high-end-brain-like chip applied at the driverless field [169–171].

Except for the above two, other biological behaviors are also demonstrated in artificial synapses and neurons. For example, sleep-wake cycle autoregulation is simulated in neural circuit of Bi_2_O_2_Se synapse reported by Zhang et al. [172]. However, more biologically intelligent behaviors will need to be developed in neuromorphic devices.

## 6. Conclusions and prospects

In the past decade, neuromorphic devices represented by memristors have demonstrated the ability to mimic brain function from synapse and neuronal action to some intelligent behaviors. The memristor displays extremely similar features with biological synapse, as its resistance depends on the history of applied voltage and current. For these reasons, most of the synaptic plasticity modes are demonstrated in the neuromorphic devices, including EPSC, IPSC, STSP, LTSP, PPF, PPD, LTP, LTD, STDP and SRDP. Arrays of synaptic devices and neural circuits are used to simulate the neuronal functions such as the information processing and integration. Specially, artificial neurons consisting of synapse (memory) and sensor element have successfully simulated the sensory nervous systems like retina, tactile, auditory, olfactory and nociceptive nervous systems. Meanwhile, the artificial synapse and neuron circuit can be applied to the simulation of biological behavior, such as the Pavlov’s dog experiment.

Even so, some challenges still trouble the development of the neuromorphic devices.

(1) *Neuroscience*. Nowadays, our understanding of the brain is still superficial. It is not fully understood how memory is stored and recovered in our brains. Development of the neuromorphic devices is inseparable from the knowledge of the original biological model.

(2) *Materials*. As mentioned above, the materials used in neuromorphic devices are various and none of these materials stands out. Similar to silicon, which is widely used in modern electronics, it is necessary to develop one or two key materials for neuromorphic devices.

(3) *Integration*. A high-power neuromorphic device should not only integrate more components but also have more functions. Neuromorphic devices should progress from memory to in-memory computing and to sensor-in-memory computing, and from passive receiving of information to active decision-making for a smart system.
